# Improving the completeness of public metadata accompanying omics studies

**DOI:** 10.1186/s13059-021-02332-z

**Published:** 2021-04-15

**Authors:** Anushka Rajesh, Yutong Chang, Malak S. Abedalthagafi, Annie Wong-Beringer, Michael I. Love, Serghei Mangul

**Affiliations:** 1 Department of Pharmacology and Pharmaceutical Sciences, University of Southern California, Los Angeles, CA 90089 USA; 2 Genomics Research Department, King Fahad Medical City and King Abdulaziz City for Science and Technology, Riyadh, Saudi Arabia; 3 Department of Clinical Pharmacy, University of Southern California, Los Angeles, CA 90089 USA; 4 Department of Biostatistics, University of North Carolina at Chapel Hill, Chapel Hill, NC 27516 USA; 5 Department of Genetics, University of North Carolina at Chapel Hill, Chapel Hill, NC 27514 USA

Over the last decade, there have been continuous efforts to improve the sharing of genomics data to allow researchers to freely access data across a wide range of phenotypes. Open omics data is widely available on searchable public repositories, ensuring that datasets are not just available but also easily discoverable [1]. Availability of this data allows for effective secondary analysis which in turn may accelerate novel biomedical discoveries. Secondary analysis comprises research techniques for analyzing data that has been collected prior to defining the current hypothesis. Using public repositories to share data substantially simplifies discovering and accessing datasets of interest, as one has scalable, programmatic access to a large number of studies. But in order for the value of publicly available omics data to be fully realized, it should be annotated appropriately. Annotation of metadata includes fully describing the sample type, procedure of collection, extraction and assay methods, and relevant clinical phenotypes. For processed or summarized data, metadata also includes aspects of the computational pipeline such as annotation (genome build, gene annotation provenance and release number), software arguments, and software versions. Lack of complete annotations may negatively impact follow-up studies aiming to reuse the omics data [2, 3].

Fair and ethical data sharing provides a firm edifice upon which the scientific research community is built. The biomedical community makes a concerted effort to share omics data but lacks consistency among researchers to ensure that metadata accompanying raw omics data is complete and fully available. Existing literature has explored how sharing of data should be FAIR—Findable, Accessible, Interoperable and Reusable [4]—and has considered accuracy, completeness, and consistency as three vital parameters to assess the quality of available metadata, although the degree to which the research community follows these principles to ensure completeness and accuracy of public metadata accompanying omics studies is currently unknown. The incompleteness of metadata and improper annotation compromises the ability to reproduce results of the original study. Public data sharing accompanied by fully described metadata allows available omics data to be effectively leveraged to accelerate novel biomedical discoveries [5], when both raw omics data and metadata are present in a standardized format. A standardized format implies that the metadata would be classified into specific well-defined categories and that there would be a predetermined minimum number of clinical phenotypes to be shared while submitting data to a public repository, for example age, sex, ancestry, and tissue type. It is imperative to note that here, we use the term “ancestry” to imply self-reported ancestry. We avoid using the word “race” because of how this term has been wrongfully used socially, as well as in medical research. “Race”, “ethnicity”, and “ancestry” are often used interchangeably but do have a difference. “Race” is an ambiguous term that has been used in varying contexts politically and culturally to (often) stereotype a particular group of people. “Ethnicity” refers to a group of people hailing from a similar cultural background while “Ancestry” can denote genetic or self-reported ancestry [6]. More populations involved in the study would mean that more variables need to be accounted for and controlled. This may be why there is a persistent European bias in GWAS, owing to logistical, systemic, and historical factors [7]. There are no fixed variables that define the term “race,” and so in this context, it would be inappropriate to use such a rigid term for this analysis.

To illustrate the completeness of metadata accompanying open omics studies, we performed a systematic assessment of completeness of public metadata accompanying open transcriptomics data of patients with sepsis and corresponding controls. In our analysis, we carefully examined 3125 transcriptomic samples across 29 transcriptomics-based sepsis studies. To estimate the completeness of metadata both in the corresponding publications and the public repositories, we first referred to a comprehensive analysis for sepsis mortality prediction [8], in which the authors had obtained complete metadata from researchers of the original sepsis studies. For the cohorts that were not included in the comprehensive analysis, we contacted the researchers owning the data, asking them for the corresponding metadata. In a few cases where we were unable to obtain the required information from the authors, we assumed that they had complete information about all clinical phenotypes under analysis. This metadata directly obtained from the researchers were considered as complete and was compared to the metadata available in the publication and public repositories. We examined nine clinical phenotypes: the disease condition, age, sex, tissue type, country of residence of the patient, ancestry, clinical tests (severity of the disease), organism, and mortality. We found that on average, 65% of clinical phenotypes were shared in the publication and/or public repository. We observed a large variability in the completeness of reported clinical phenotypes. First, the percentage of reported clinical phenotypes varied from 83.3% for the most complete study to 38.9% for the least. Next, we found that the most reported clinical phenotypes were organism and tissue type (100%) while the least reported was ancestry (22.4%).

There were some marked inconsistencies between the clinical phenotypes reported in the publication and the repository, with 35% of the information being lost from the publication to the repository. The most reported clinical phenotypes on publications were the disease condition, organism, and tissue type, each being consistently reported across all studies, while this was also the case for the latter two on public repositories. The least reported clinical variable on publications was ancestry (37.9%), while on the repositories, it was the country of residence (3.4%). Overall, apart from tissue type and organism, the least discrepancy was observed for reporting the disease condition, with 100% being reported on publications and 82.8% reported on the repositories. On the other hand, the largest difference between publications and repositories was observed for the country of residence of the patient. While the country is mentioned on 89.7% of the publications, it was reported by a mere 3.4% on public repositories. Interestingly, although both platforms were not complete in terms of the metadata they share, we have found that about 45.7% of the total data was lost between publication and that shared on public repositories. It is essential to make a conscious effort to share data and the corresponding metadata on both platforms. When data is shared only in the publication, it becomes infeasible to manually parse the text of publication to extract the relevant metadata, especially when looking at a large number of studies. Public repositories play a significant role in sharing of both raw omics data and corresponding metadata allowing instant access to millions of diverse datasets across various diseases and phenotypes. In contrast, a publication format is not well suited to share the metadata as metadata scraping is a laborious and error- prone approach, requiring the manual search and extracting of the clinical phenotypes from the text of publication. Public repositories thus have a very important role in ensuring data sharing and we therefore need rigorous standards in terms of how the metadata is shared. Having standardized metadata will allow researchers to obtain complete and consistent information across all platforms, whether it is from a publication or from a repository.

Open, freely accessible, and standardized metadata in an easy-to-use format is the key for reproducibility of the findings reported in the original publication. Completeness of metadata enables reusing open omics data and undoubtedly offers multifarious benefits to the scientific community. Broader and more complete sharing of omics data and corresponding metadata will promote extensive research especially in cases where the scientists may find it expensive to generate new data [9].

It was a substantial effort in the biomedical community to address poorly structured and incomplete metadata associated with the open omics data. For example, a tool called MetaSRA has been developed to standardize the raw (unstandardized) metadata accompanying experiments on the Sequence Read Archive (SRA) [10]. MetaSRA directly derives information from the available omics metadata and does not extract it from the publication or from the raw omics data itself. In general, it is possible to infer certain phenotypes directly from omics data (e.g., sex, genetic ancestry) [11]. Such omics-derived phenotypes promise to complement standard metadata or serve as quality controls to capture human-generated errors in reported metadata. To address the inconsistencies between the clinical phenotypes reported in the publication and the repository, natural language processing (NLP) methods show promise to extract metadata in a standardized format directly from the text of the publication. Recently, METAGENOTE has been publicized as a web portal that helps in annotation of metadata and streamlining the submission process to SRA [5]. It would be extremely beneficial to the biomedical community if a similar platform is developed for the other repositories as well. Efforts are also being made to standardize computationally derived metadata [11], with attention paid to creating a database that has very structured information about any transformations being made, and bidirectional links to repositories where the metadata is stored.

There is an emerging need to establish a standard for reporting metadata to ensure well-rounded and complete sharing of metadata with the broad scientific community. A significant effort has been made in the biomedical community to standardize metadata. The Genomic Standards Initiative has laid down minimum requirements for reporting nucleotide sequences (MIxS) [12] and metadata, and similarly, the Microarray Gene Expression Database Group has recommended requirements for describing transcriptomic data (MIAME/Plant) [13]. Researchers have suggested similar guidelines to be set for plant phenotypic data [14], and the same initiative is critically needed for biomedical research. The Genomic Standards Consortium facilitates data integration, discovery, and comparison by establishing international standards [15]. These recommendations suggest what phenotypes should be shared along with the omics data; however, there remains a lack of formal guidelines on the format that the metadata needs to be shared in [11].

There are several barriers which may prevent scientists from sharing clinical phenotypes associated with omics data. First, it may be due to the study design proposed by the research group to the Institutional Review Board (IRB). Once proposed to and approved by the IRB, the study protocol cannot be altered. This means that if a certain clinical variable has been neglected from the proposal, it cannot be reported in the publication or repository even if results are available. For example, if ancestry has not been taken into consideration in a study design because it has been performed on genotypically and phenotypically similar individuals, it cannot be reported later as a clinical variable in the published study. A potential solution to this would be that the IRB itself establishes a minimum requirement checklist for omics studies that spans across all possible clinical phenotypes that could be reported. This checklist should be envisioned by keeping in mind not only the present research group, but also the subsequent groups that may benefit from reusing the data. Another barrier to sharing of clinical phenotype information may be that the individuals have been de-identified while publishing the data, for the purpose of maintaining study subject confidentiality, and the researchers will not be able to contact them for more information. Lastly, there is a somewhat cultural barrier, where researchers may weigh the value of sharing the metadata with the community against other considerations.

We recognize a need to draw the attention of the biomedical community towards an emerging need for structuring metadata to combat the current situation wherein substantial portions of metadata accompanying open omics datasets are incomplete and often poorly annotated. It is imperative to make shared metadata as structured as possible, with unstructured text elements used only when a structured representation is not supported by the repository [8]. Moreover, data from studies and their corresponding metadata should be deposited to public repositories as rapidly as possible. This will prevent data from being lost between the publication and the repository by minimizing the risk of the researchers not being able to track their datasets and the associated metadata over time [8]. We would like to open a wide discussion of the potential solution to bridge the gap between raw omics data and metadata by suggesting the benefit of having a fixed yardstick that describes the minimum set of clinical phenotypes required to be collected and disclosed by the researcher while submitting the data, and a standardized format to be adopted by the biomedical community.

## Data Availability

All supporting material for this analysis can be found at https://github.com/Mangul-Lab-USC/metadata_sepsis.
